# Comprehensive Analyses of Bone and Cartilage Transcriptomes Evince Ion Transport, Inflammation and Cartilage Development-Related Genes Involved in Chickens’ Femoral Head Separation

**DOI:** 10.3390/ani12060788

**Published:** 2022-03-20

**Authors:** Iara Goldoni, Adriana Mércia Guaratini Ibelli, Lana Teixeira Fernandes, Jane de Oliveira Peixoto, Ludmila Mudri Hul, Maurício Egídio Cantão, João José de Simoni Gouveia, Mônica Corrêa Ledur

**Affiliations:** 1 Programa de Pós-Graduação em Ciências Veterinárias, Universidade Estadual do Centro-Oeste, R. Salvatore Renna, 875, Guarapuava 85015-430, PR, Brazil; iara_goldoni@hotmail.com (I.G.); jane.peixoto@embrapa.br (J.d.O.P.); ludhul@yahoo.com.br (L.M.H.); 2 Embrapa Suínos e Aves, Rodovia BR 153, km 110, Concórdia 89715-899, SC, Brazil; lanatf@yahoo.fr (L.T.F.); mauricio.cantao@embrapa.br (M.E.C.); 3 Programa de Pós-Graduação em Ciências Veterinárias no Semiárido, Universidade Federal do Vale do São Francisco, UNIVASF, Rodovia BR 407, 12 Lote 543, Petrolina 56300-000, PE, Brazil; joao.gouveia@univasf.edu.br; 4 Programa de Pós-Graduação em Zootecnia, UDESC-Oeste, Rua Beloni Trombeta Zanin, 680E, Chapecó 89815-630, SC, Brazil

**Keywords:** gene expression, SNPs, femoral head necrosis, inflammatory response, integrated analysis

## Abstract

**Simple Summary:**

Femoral head necrosis (FHN) and other locomotor problems cause severe impacts on the poultry industry due to huge economic losses and reduced animal welfare. Femoral head separation (FHS), the initial phase of FHN, is usually a subclinical condition characterized by the detachment of articular cartilage from the bone. In this study, we aimed to identify genes and biological processes involved with FHS in broilers. A better understanding of the FHS molecular mechanisms can help to develop strategies to reduce this condition in chickens. Here, we described several genes that have their expression altered in the articular cartilage and femur when normal and FHS-affected animals were compared. Furthermore, genetic variants were found differing between the studied groups. Therefore, performing an integrated analysis of these datasets, we were able to detect genes and variants related to FHS in chickens. Some of them, such as *SLC4A1*, *RHAG*, *ANK1*, *MKNK2*, *SPTB*, *ADA*, *C7* and *EPB420* genes were highlighted and should be further explored to validate them as candidates to FHS and FHN in chickens and possibly in humans.

**Abstract:**

Femoral head separation (FHS) is usually a subclinical condition characterized by the detachment of articular cartilage from the bone. In this study, a comprehensive analysis identifying shared and exclusive expression profiles, biological processes (BP) and variants related to FHS in the femoral articular cartilage and growth plate in chickens was performed through RNA sequencing analysis. Thirty-six differentially expressed (DE) genes were shared between femoral articular cartilage (AC) and growth plate (GP) tissues. Out of those, 23 genes were enriched in BP related to ion transport, translation factors and immune response. Seventy genes were DE exclusively in the AC and 288 in the GP. Among the BP of AC, the response against bacteria can be highlighted, and for the GP tissue, the processes related to chondrocyte differentiation and cartilage development stand out. When the chicken DE genes were compared to other datasets, eight genes (*SLC4A1*, *RHAG*, *ANK1*, *MKNK2*, *SPTB*, *ADA*, *C7* and *EPB420)* were shared between chickens and humans. Furthermore, 89 variants, including missense in the *SPATS2L*, *PRKAB1* and *TRIM25* genes, were identified between groups. Therefore, those genes should be more explored to validate them as candidates to FHS/FHN in chickens and humans.

## 1. Introduction

The poultry industry has experienced huge growth in recent decades, due to the advances in new research and technologies, which has brought benefits to the industry and producers. Chicken meat is one of the most consumed proteins in the world, due to the low cost, for being considered cost-effective and healthy, and because it is exempted from cultural impediments [1]. The constant investment in genetic improvement, nutrition and management has generated a better performance of the broilers, with slaughter age reduction, better feed conversion and less water consumption, always ensuring the animal welfare [2].

The genetic breeding programs have brought numerous benefits for poultry production, such as the increased body weight, greater thoracic musculature, and reduction of abdominal fat. In the last 50 years, chicken growth rates have risen more than 300%, from 25 g per day to approximately 100 g per day [3,4]. However, some negative consequences, such as locomotor problems and immunological and metabolic disorders have appeared due to the selection for fast-growing [3,5].

Locomotor problems cause severe impacts on the poultry industry, affecting about 6% of the animals in commercial flocks, which results in huge economic losses [6]. Furthermore, animals with bone disorders have their welfare affected because they cannot eat and drink water properly, being susceptible to opportunistic agents [7]. The bacterial chondronecrosis with osteomyelitis (BCO), also known as femoral head necrosis (FHN), is the most prevalent leg disorder in broilers [8]. The real incidence of FHN in broilers is difficult to estimate since most of the lesions often remain subclinical, and its etiological basis is not fully understood [9]. However, some authors have shown 20% of FHN incidence in chickens [10]. FHN occurs in the proximal part of the chickens’ femur head, starting with the femoral head separation (FHS), which consists in the separation of the growth plate (GP) from the articular cartilage (AC) [9,11]. This occurs due to the high growth rates and the mineralization deficiency of the chondrocytes that cause damage to the bone, which could allow for the colonization by opportunistic bacteria featuring the BCO [12].

Since the etiology of FHN remains unclear, studies are being conducted to clarify its pathogenesis and the molecular mechanisms involved, and to discover genes with a potential role in triggering FHS and subsequent BCO in the head of the femur and tibia [10,13,14,15,16,17]. Although most of the previous studies describe biological processes (BP) involved with this condition in bone tissue, there are no studies evaluating gene expression profiles between bone and articular cartilage of normal and FHS-affected chickens. The femur GP or physis is a point of huge stress during the broilers growing phase. The GP tissue presents a cartilaginous matrix and sequential layers of long columns of chondrocytes in different maturation stages [12]. Above the GP is the AC that consists of chondrocytes and extracellular matrix (ECM), which is mainly composed by proteoglycan, glycosaminoglycan, and collagen fibers [18]. The understanding on how these tissues are interacting might be one way to improve the knowledge of molecular mechanisms involved with FHS. Therefore, in this study, the femoral growth plate and articular cartilage transcriptomes from normal and FHS-affected broilers were compared to identify common and exclusive molecular mechanisms and differentially expressed (DE) genes between groups, and polymorphisms highlighting possible new candidate genes involved with FHS in chickens.

## 2. Material and Methods

Transcriptomes used in the present study were obtained from a project that evaluated femoral head separation (FHS) in the growth plate and articular cartilage, published respectively by Peixoto et al. [15] and Hul et al. [17], which are available in the SRA database BioProject # PRJNA350521 for articular cartilage (AC) and PRJNA352962 for growth plate (GP). Briefly, 16 AC and GP samples from 35-day-old male broilers (Cobb500), previously characterized as normal (4 AC and 4 GP) or FHS-affected (4 AC and 4 GP), were used in this study. At the time of collection, the normal samples had a good adhesion between the AC and GP, and the FHS-affected samples had the AC easily detached from the GP. For the normal group, the AC was separated from the GP using a scalpel. These tissues were submitted to RNA extraction using Trizol and a cleanup using RNeasy mini kit (Qiagen, Hilden, Germany). RNA quality was assessed in 1% agarose gel and in Agilent 2100 Bioanalyzer (Agilent, Santa Clara, CA, USA). Samples with an RNA integrity number (RIN) higher than 8 were used to construct the RNA-Seq libraries. Eight samples from each tissue (4 normal and 4 FHS-affected), were prepared for RNA sequencing. For GP, the libraries were prepared from 2 µg of RNA using the “TruSeq RNA Sample Prep Kit v2” (Illumina, San Diego, CA, USA), while for AC the “TruSeq Total Stranded Sample Preparation” (Illumina, San Diego, CA, USA) kit was used. Libraries were sent to the Functional Genomics Center, ESALQ, University of São Paulo, Piracicaba, São Paulo State, Brazil, for sequencing in Illumina HiSeq2500 equipment (2 × 100 bp, Illumina, San Diego, CA, USA), with samples of each tissue in a different lane.

### 2.1. Bioinformatics, Differential Expression Analyses and Functional Annotation

The baqcom pipeline (https://github.com/hanielcedraz/BAQCOM, accessed on 31 December 2021) was used for quality control (QC) and mapping. QC for each sequencing file was performed with the Trimmomatic v. 0.38 [19] to remove short reads (<70 bp), low-quality reads (QPhred < 20) and adapter sequences. AC and GP sequences were mapped against the chicken reference genome (GRCg6a, Ensembl 101) using the STAR software v 2.7 [20], and reads were counted with HTSeq-counts [21]. The EdgeR package [22] was used to identify the DE genes between normal and affected groups in each tissue separately, considering differentially expressed those with false discovery rate (FDR) ≤ 0.05 after correcting for the Benjamini–Hochberg (BH) multiple tests [23]. Positive and negative log2 fold-change corresponded to DE genes upregulated or downregulated, respectively, in FHS-affected compared to the normal group. A heatmap was generated to visualize patterns of expression across samples from both tissues using R. The expressed genes were annotated using the Biomart database (https://www.ensembl.org/biomart, accessed on 31 December 2021). Visualization and Integrated Discovery (DAVID 6.8) [24] and Panther (http://pantherdb.org/, accessed on 31 December 2021) [25] databases were used for assessing functional profiles of DE genes based on the biological processes (BP), cellular components (CC), molecular functions (MF) and metabolic pathways categories of gene ontology (GO). The enrichment analyses were performed using the chicken genome information available in those databases.

### 2.2. qPCR Validation

A relative quantification using qPCR was performed to confirm the expression profile of eight chosen DE genes in the GP using eight samples from the four normal and four from the FHS-affected group. The genes selected were solute carrier family 4 member 1 (*SLC4A1*), Rh- associated glycoprotein (*RHAG*), ankyrin 1 (*ANK1*), MAP kinase interacting serine/threonine kinase 2 (*MKNK2*), spectrin beta, erythrocytic (*SPTB*), adenosine deaminase (*ADA*), complement C7 (*C7*) and erythrocyte membrane protein band 4.2 (*EPB42*). Gene sequences were downloaded from Gallus gallus on Genebank (http://www.ncbi.nlm.nih.gov/gene/, accessed on 31 January 2022) and Ensembl (www.ensembl.org, accessed on 31 January 2022). Primers were designed in exon–exon junctions using the Primer-Blast online tool [26], and their quality was evaluated in the NetPrimer online software (http://www.premierbiosoft.com/netprimer/, accessed on 31 January 2022) (Table 1). For AC, the analysis was also performed in eight samples for five genes (*SPTB*, *MKNK2*, *SLC4A1*, *C7* and *EPB42*), since the RHAG, ANK1 and ADA genes have already been confirmed in the analyzed population by Hul et al. [17]. Ribosomal protein 4 (RPL4) and Ribosomal protein 30 (RPL30) genes were used as reference for GP, and Ribosomal protein 5 (RPL5) and Ribosomal Protein Lateral Stalk Subunit P1 (RPLP1) for AC, as described by Peixoto et al. [15] and Hul et al. [17], respectively. The cDNA was synthetized using SuperScript III First-Strand Synthesis SuperMix (Invitrogen, Waltham (MA), USA), and qPCR reactions were carried out in QuantStudio 6 (Applied Biosystems, Waltham (MA), USA) equipment. The reactions had a final volume of 15 μL with 1×GoTaq qPCR Master Mix with BRYT Green (Promega, Madison (WI), USA), 0.13 µM of each primer and 2 μL of cDNA. Cycle threshold (Ct) mean for each replicate sample was obtained and normalized using the geometric mean of the reference genes, calculating 2^−ΔCt^ [27]. Statistical analyses for group comparisons were performed using Mann–Whitney–Wilcoxon test in the R environment, considering DE as the genes with *p* ≤ 0.05.

### 2.3. Integrated Analysis

To identify the main molecular mechanisms involved in FHS, the common and exclusive DE genes in the AC and GP datasets were obtained, and the David [24] database was used to find BP involved with FHS based on the gene ontology database. The REVIGO tool [28] was used to reduce and highlight the most abundant BP in our data. Furthermore, gene networks with the common DE genes between the two tissues and those obtained with DE genes only detected in AC or GP were constructed using the STRING database [29].

Furthermore, we used the Gene Expression Omnibus (GEO) database (https://www.ncbi.nlm.nih.gov/gds, accessed on 9 November 2020) to search datasets with the terms “femoral head necrosis” and “epiphysiolysis” to better understand the role of the main DE genes involved with FHS across different species. This search returned two main datasets: identification of potential biomarkers for improving the precision of early detection of steroid-induced osteonecrosis of the femoral head (GSE123568) and gene expression profile of hip cartilage with necrosis of femoral head (GSE74089). The first one evaluated the gene expression on blood from 40 patients (10 normal and 30 affected with femoral head necrosis) and the second one, the hip cartilage in 24 patients (12 normal and 12 affected with femoral head necrosis). DE genes were obtained using the GEOR tool from the GEO dataset (https://www.ncbi.nlm.nih.gov/geo/info/geo2r.html, accessed on 9 November 2020). The list of DE genes from the two datasets obtained in the GEO database and the DE genes found in the GP and AC from our study were submitted to the InteractiVenn (http://www.interactivenn.net/, accessed on 10 November 2020) [30] to find common DE genes among the experiments.

### 2.4. Polymorphism Identification Using the RNA-Seq Data

The Genome Analysis Tool kit 3.6 (GATK) [31] was used for polymorphism identification. We followed the best practices guidelines standard parameters for transcriptome variant analysis available on the GATK website (https://gatk.broadinstitute.org/, accessed on 1 February 2021). The Picard tools 2.5 (https://broadinstitute.github.io/picard/index.html, accessed on 17 May 2017) was used to generate the genome index, to assign read groups and mark duplicates. In the GATK, the CIGAR strings determination (SplitNCigarReads), qualities reassigning mapping, base recalibration, variants calling, and filtering were performed. To minimize the false positive variants, the following filters were used in GATK to filter and select variants: FS > 30.0, MQRankSum < −12.5, SNPcluster considering 3 variants in a 35 bp window, QD < 5.0, MQ < 50.0, GQ < 5.0, QUAL ≥ 30.0, ReadPosRankSum < −8.0 and DP ≥ 100.0. Furthermore, variants with less than 10 reads per sample were removed from the downstream analysis. Once the polymorphisms were identified in the GP and AC datasets, a filter was performed to obtain the variants that differed between normal and FHS-affected groups. The Variant Effect Predictor (VEP) tool [32] was used for variant annotation, effect and consequence predictions using the *Gallus gallus* genome (GRCg6a) with the Ensembl 103 annotation database version. Furthermore, the Stringdb [29] and EnrichR [33,34] databases were used to verify the interactions between DE genes and variants identified in genes.

## 3. Results

### 3.1. RNA-Sequencing, Mapping and Characterization

The RNA sequencing generated about 345 million paired end reads for all samples (N = 16), and after the quality control, approximately 306 million reads (88.78%) remained for further analyses (Supplementary File S1: Table S1), with an average of 16,355,490 reads for GP and 26,819,697 for AC. A clear separation between the samples from the two tissues, as well as between the normal and affected groups can be verified in the heatmap (Supplementary File S2: Figure S1). As expected, most of the genes were characterized as protein coding (95%), and the remaining 5% were classified in *IncRNA*, pseudogenes, miRNAs and other noncoding RNAs (Table 2).

### 3.2. Differentially Expressed Genes in AC and GP Transcriptomes

Using the Ensembl annotation 101, in the AC transcriptome, 12,182 genes were identified and 106 genes were DE; as well, 99 (93.44%) were upregulated and seven (6.56%) were downregulated in the AC of the FHS-affected compared to the normal group (Supplementary File S1: Table S2). Regarding the GP tissue, 12,632 genes were expressed, where 324 were DE between the analyzed groups. From those, 174 were upregulated (53.7%) and 150 (46.3%) downregulated in the affected compared to the normal group (Supplementary File S1: Table S3).

Comparing the DE genes between the AC and the GP, 36 DE genes were common to both transcriptomes (Table 3). From these 36, 33 were upregulated and three were downregulated in the FHS-affected group in both transcriptomes. From the 70 DE genes remaining in the AC data, four were downregulated and 66 were upregulated in the FHS-affected broilers (Table S2). Finally, 141 genes were upregulated and 147 were downregulated in the affected group when considering the DE genes exclusive to the GP tissue (Table S3).

**Figure 1:**
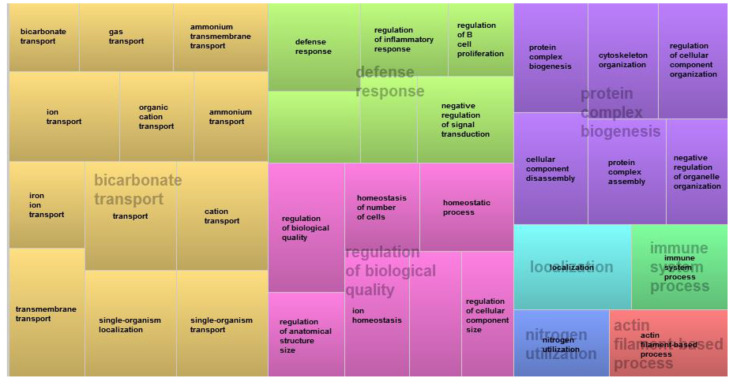
Main biological process involved with FHS considering the DE genes shared between articular cartilage and femoral growth plate using the REVIGO tool.

The enrichment analysis performed based on the common DE genes between the two tissues showed that the main biological processes found were related to ion transport, inflammatory and defense response, homeostasis, protein biogenesis and immune response processes (Figure 1).

The most relevant BP for the AC tissue was the defense response to bacterium, cell chemotaxis and regulation of cell motility (Table S4). Conversely, the regulation of lipid metabolic process, cytokine-mediated signaling pathway, T cell activation, inflammatory response, interleukin-1 beta production, small GTPase-mediated signal transduction, and negative regulation of chondrocyte differentiation and cartilage development were the BP most prominent on GP tissues (Table S5).

**Figure 2:**
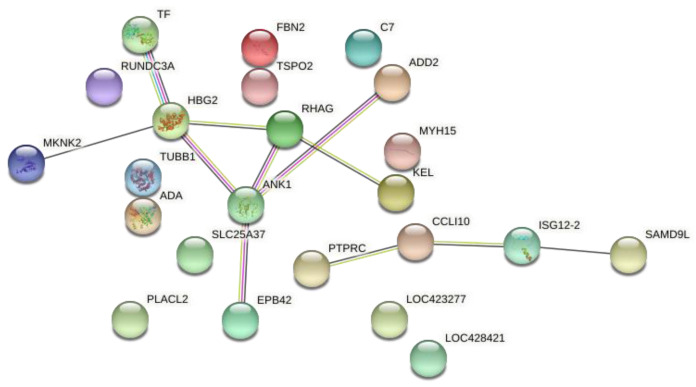
Gene network of the common DE genes between the articular cartilage and femoral growth plate tissues from normal and FHS-affected groups using STRING. Colored circles represent genes, and lines represent the predicted interactions between genes.

A gene network was constructed based on the 36 common DE genes. From those, 23 were recognized by Stringdb tool, where it was possible to observe a main network of grouped genes related to ion transport and translation factors (Figure 2). The transferrin (*TF)* gene is involved in antimicrobial activity, while some genes such as Hemoglobin subunit gamma-2 (*HBG2)* and Rh-associated glycoprotein (*RHAG)* are involved in oxygen and ion transport. Another group connected the *PTPRC*, *CCL10*, *ISG12-2* and *SAMD9L* genes that are most related to the immune response BP (Figure 2).

**Figure 3:**
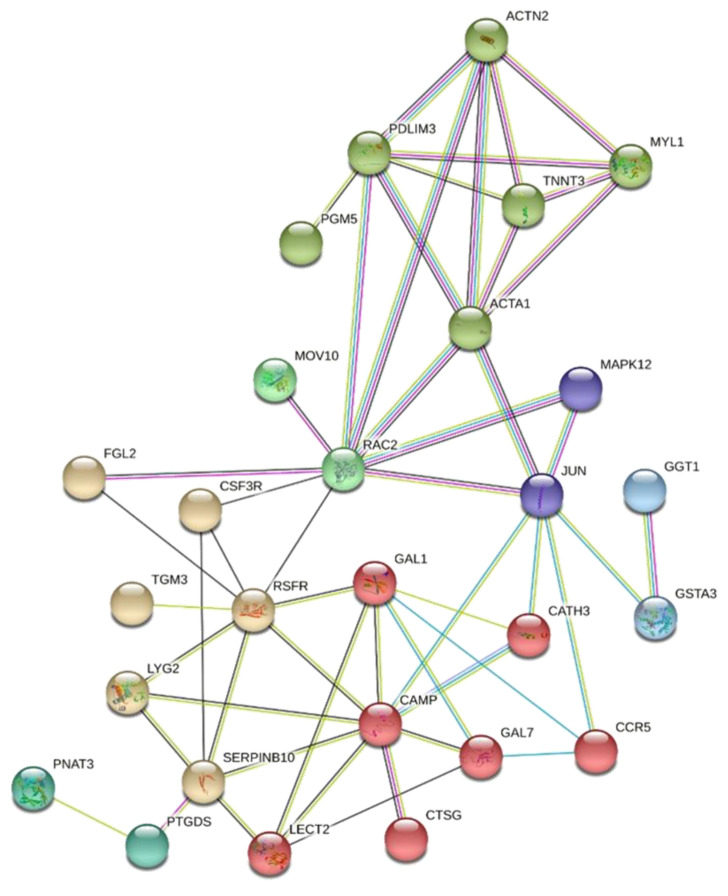
Gene network of unique DE genes in the articular cartilage from normal and FHS-affected broilers using STRING. Colored circles represent genes with similar functions or acting in similar pathways, and lines represent the predicted interactions between genes.

**Figure 4:**
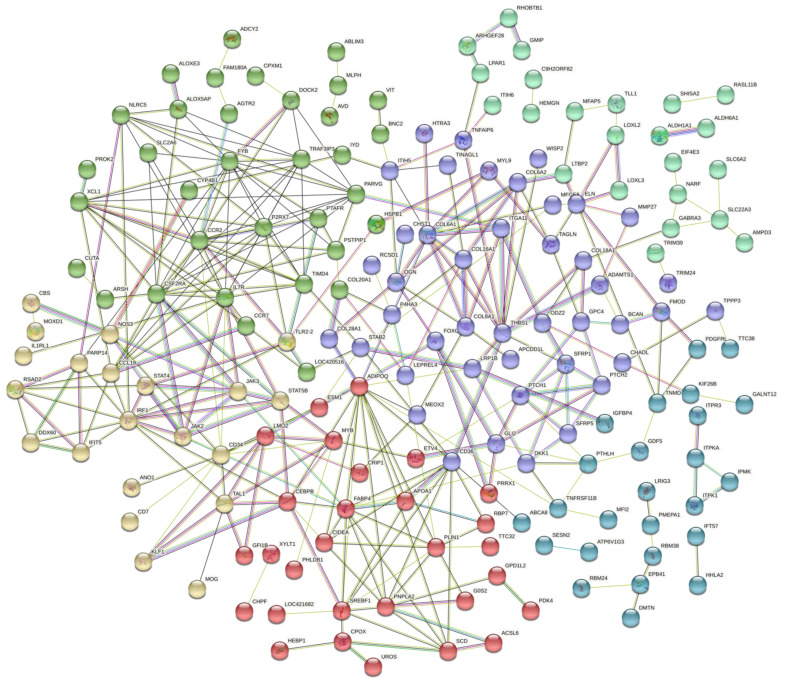
Gene network of unique DE genes in the femoral growth plate of normal and FHS-affected broilers using STRING. Colored circles represent genes with similar functions or acting in similar pathways, and lines represent the predicted interactions between genes.

Another two gene networks were constructed based on the DE genes exclusive for AC or for GP. For AC, 61 genes were recognized by the Stringdb tool, which highlighted a group of genes related to the response against bacteria, with all genes involved in this BP being more expressed in the FHS-affected group (Figure 3). The second network was constructed with 266 GP DE genes, and the processes related to chondrocyte differentiation and cartilage development stood out, grouping genes that had the lowest expression in the affected group (Figure 4).

### 3.3. qPCR Validation

**Figure 5:**
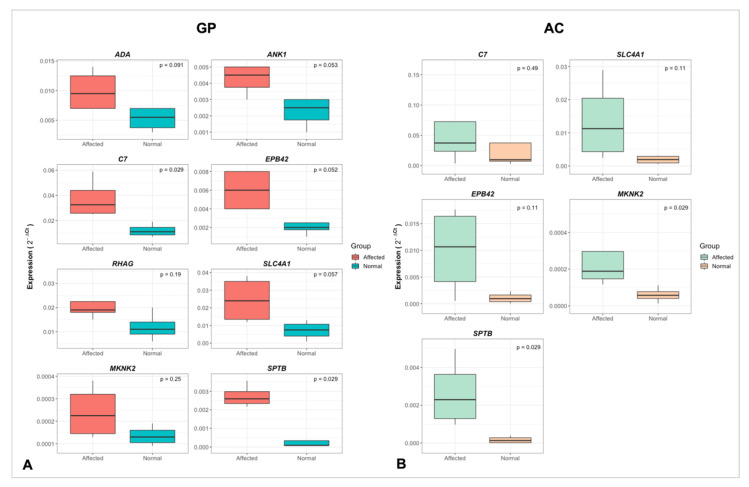
Expression profile (2^−ΔCt^) and statistical significance between normal and FHS-affected broilers obtained with qPCR for GP (**A**) and AC (**B**) for the candidate genes evaluated.

In the qPCR analysis, from the eight candidate genes evaluated in GP, four (*C7*, *EPB42*, *MKNK2* and *SPTB*) were DE. The MKNK2 and SPTB genes were also DE in the AC. Furthermore, all evaluated genes were upregulated in the FHS-affected group (Figure 5A,B), having the same expression profile and therefore confirming the RNA-Seq results.

### 3.4. Comparison of the DE Genes from AC and GP Tissues with Different Datasets

**Figure 6:**
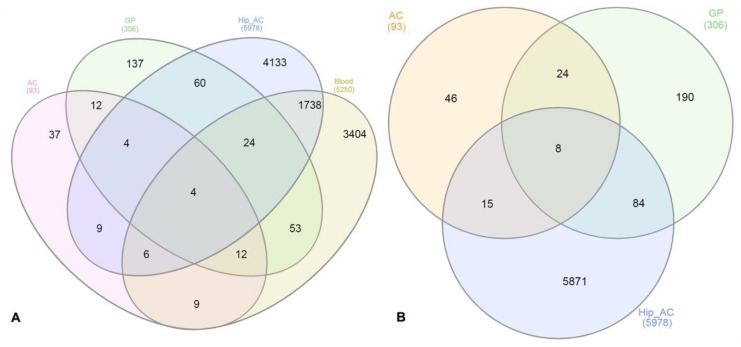
(**A**) Venn diagram constructed with DE genes from normal and femoral head necrosis and/or FHS-affected samples in four evaluated datasets. AC: chicken femoral articular cartilage; GP: chicken femoral growth plate; Hip_AC: human articular cartilage; Blood: human peripheral blood. (**B**) Venn diagram constructed with DE genes from normal and femoral head necrosis and/or FHS affected samples in three evaluated datasets. AC: chicken femoral articular cartilage; GP: femoral growth plate; Hip_AC: human articular cartilage.

After the differentially expressed analysis, we compared the DE genes from the AC and GP tissues with other datasets obtained in the GEO database, as previously described. According to the GEOR analysis, the comparison performed between normal and FHN-affected individuals (GSE123568) evinced 5250 DE genes in the blood of those individuals, while 5978 DE genes were found in the hip cartilage of normal and FHN-affected individuals (GSE74089). Considering these four datasets, four genes were DE in all studies: *SLC4A1* (Solute Carrier Family 4 Member 1), *EPB42* (Erythrocyte Membrane Protein Band 4.2), *SPTB* (Spectrin Beta, Erythrocytic) and *ANK1* (Ankyrin 1) (Figure 6A), all of them related to erythrocyte and ion transport functions.

We also performed a comparison using DE genes from cartilage (chicken and human) and bone datasets to verify the presence of shared genes in the tissues affected by FHN/FHS. In this comparison, eight shared genes were found: the four previously described (*SLC4A1*, *EPB42*, *ANK1* and *SPTB)* and four additional genes: *CCL26* (Chemokine (C-C motif) ligand 26), *ADA* (Adenosine Deaminase), *SLC25A37* (Mitoferrin-1) and *MKNK2* (MAPK Interacting Serine/Threonine Kinase 2), all involved with immune response (Figure 6B).

### 3.5. Variant Identification and Annotation

**Figure 7:**
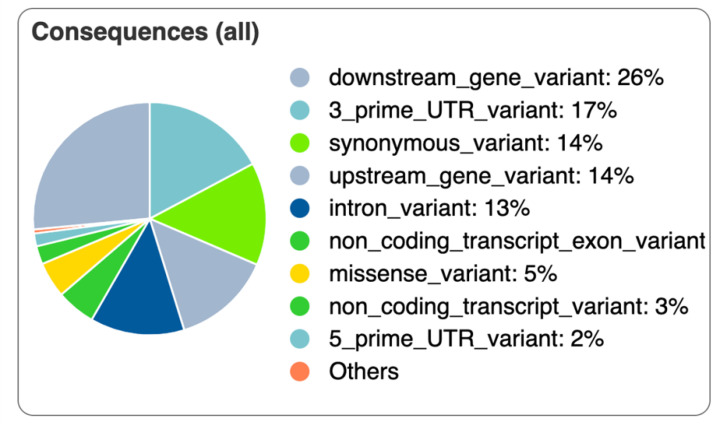
Consequences of all variants identified the articular cartilage and growth plate transcriptomes between normal and affected broilers using the VEP tool.

A total of 89,823 variants (SNPs and InDels) were found in the analyzed samples with the GATK tool. Out of those, 89 differed between the normal and FHS-affected groups (Supplementary File S1: Table S6). These 89 variants were processed in the VEP tool and approximately 3% from all of them (three) were first described in the current study, while 86 (~97%) have already been identified in chickens. One of the three novel SNPs was located in the 5′ UTR region of the *NET1* (Neuroepithelial Cell Transforming 1) gene, another SNP overlapped the downstream region of two genes, CCM2 Scaffold Protein (*CCM2)* and NAC Alpha Domain Containing (*NACAD*, ENSGALG00000050931—predicted), and the other was a missense variant in the Staphylococcal Nuclease and Tudor Domain Containing 1 (*SND1*, ENSGALG00000054297—predicted). Variants were mainly classified as downstream gene variant, synonymous variant, upstream gene variant, intron variant, 3′ prime UTR variant and missense variants (Figure 7).

Out of the described coding variants, 90% was classified in synonymous variants, 10% was missense (Supplementary File S1: Table S7). Considering the four missense variants, three were previously described in the genes *PRKAB1* (rs733851944), *SPATS2L* (rs737569658) and *TRIM25* (rs314175171), with a Sorting Intolerant from Tolerant (SIFT) score of 0.36 and 0.16. The novel variant (chr1:1049560) in the *SND1* had a SIFT = 0, being predicted as deleterious.

After identifying the variants that differ between the normal and affected groups in the RNA sequences, a search was performed to verify if some of the variants were present in the DE genes in both AC and GP tissues previously found between normal and FHS-affected samples. None of the variants were found in the common DE genes; however, two polymorphisms were found in the DE genes in the GP: a SNP (rs737937191) in the downstream region of the *LOXL2* gene and the other in the amino acid position 52 of the *SND1* (ENSGALG00000054297) gene.

Some SNPs that differed between normal and affected broilers were found in genes that have already been described in genomic regions associated with related phenotypes in the chicken QTLdb (https://www.animalgenome.org/cgi-bin/QTLdb/GG/index, accessed on 31 December 2021), such as a SNP in the *RPS24* gene, located in a QTL region associated with tibia volume (135,882, Animal QTLdb) (Supplementary File S1: Table S8). Furthermore, some of the genes that have variants differing between the normal and affected groups, such as Solute Carrier Family 25 Member 3 (*SLC25A3*), Yip1 Domain Family Member 3 (*YIPF3*), DEAD-Box Helicase 55 (*DDX55)*, *Exportin 5 (XPO5)*, RNA Polymerase I And III Subunit C *(POLR1C)*, R3H Domain And Coiled-Coil Containing 1 *(R3HCC1)*, Transmembrane P24 Trafficking Protein 2 (*TMED2)*, Ras Responsive Element Binding Protein 1 (*RREB1)*, charged Multivesicular Body Protein 7 *(CHMP7)*, DNA Polymerase Eta *(POLH)* and Lysyl oxidase homolog 2 (*LOXL2*) have already been associated with waist-to-hip ratio adjusted for body mass index (BMI) in humans (Supplementary File S1: Table S9).

**Figure 8:**
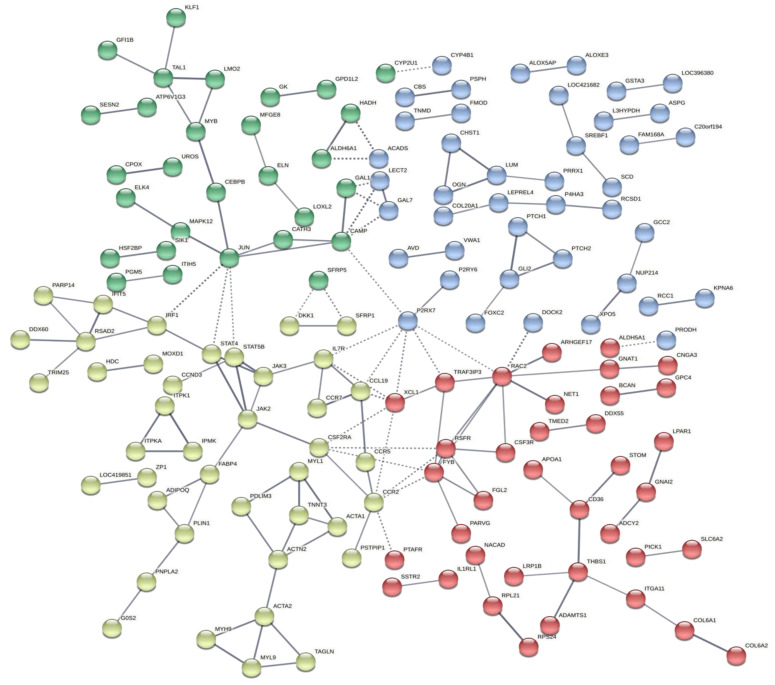
Gene network constructed using the Stringdb with DE genes and genes with SNPs differing between control and affected groups. The line thickness indicates the strength of data support. The circles represent genes, and different colors represents group of genes with similar functions or acting in similar pathways.

The interactions between the DE genes and genes with variants were verified through a gene network, where it was possible to observe that those genes have physical or functional interactions and were grouped in five main subnetworks (Figure 8). For example, the *ADA* gene was DE in AC and GP and grouped with several genes, which have SNPs differing between normal and affected groups, such as the *TRIM25*, *LUM*, *MYH9*, *RPS24* and *NACAD*.

## 4. Discussion

The mechanical stress caused by the overload of the skeleton that modern chickens sustain due to the high growth rates has been recognized as one of the main causes of locomotor problems [11,12,35]. Among these locomotor problems, the FHN/BCO is a disorder that has a large impact on the poultry production due to the huge economic losses and its negative effect on animal welfare [8,9,15]. One of the biggest issues to study FHN is its early detection, since the animals do not show clinical signs often, being visible only after slaughter [16].

There are few studies related to FHN and other bone integrity problems in chickens, especially those approaching the molecular mechanisms involved with these conditions. Previous studies from our group have shown some biological processes (BP) and genes associated with FHN/BCO in different ages and lines of chickens [14,15,16,17]. Paludo et al. [13] pointed out that the downregulated expression of *RUNX2* and *SPARC* genes may be associated with reduced vascularization and poor bone mineralization, increasing the risk of skeletal problems in chickens. The impaired collagen formation, connective tissue cell adhesion, and bone extracellular matrix (ECM) were also identified as predisposing factors for BCO and other leg disorders [14,15,16,17]. The translocation of bacteria from blood to bone has also been suggested as a secondary condition [16], whereas the bacterial profile of the microbiome in the blood of animals affected with BCO was different from healthy ones [36]. However, most of these studies focus only on the bone tissue, and there are no studies evaluating the articular cartilage and the femoral head jointly. Therefore, our study aimed to find common DE genes and BP between those tissues as well as genes exclusively DE in each tissue (AC and GP), and some of these genes were validated by qPCR (Figure 5). We also identified variants in the AC and GP transcriptomes between normal and FHS-affected broilers through an integrated analysis of the RNA sequencing datasets previously published by Peixoto et al. [15] and Hul et al. [17].

In the global transcriptome characterization of the GP and AC tissues, about 12,000 genes were expressed in both tissues. From those, 11,141 were shared and 891 and 441 genes were exclusively to the GP and AC, respectively. As expected, due to the RNA sequencing methodology used, approximately 95% of the genes were coding, although lncRNAs, miRNAS, mitochondrial and miscRNAs were also identified in the analyzed datasets (Table 2).

A total of 36 DE genes were shared by both tissues, and 91.6% of the common genes were upregulated in the FHS-affected group (Table 3). Some of those genes have already been highlighted in other studies [14,16,17]. However, when the common BPs were evaluated, most of them were related to ion transport, defense response and those related with tissue homeostasis (Figure 1).

Two main branches were observed in the common DE genes network (Figure 2): one was composed of the *TF*, *HBG2*, *ANK1*, *SLC25A37*, *EPB42*, *RHAG*, *ADD2*, *KEL*, *MKNK2* genes and another by the *PTPRC*, *CCLI10*, *ISG12-2 AND SAMD9L* genes. In general, the first group of genes are related to blood type and to cartilage regeneration, and the second is related to inflammatory response [15,37]. Moreover, a set of variants was identified differing between normal and affected groups, where none of them were located in the common DE genes. It was observed that some of these polymorphisms were in genes that are co-expressed or co-regulated with DE genes (Figure 7). The possible role of some of these genes in the FHS are discussed below.

The transferrin (*TF*) was in the main branch of the gene network and its primary function is to transport iron to tissues that require this mineral [38], being involved in defense against systemic infection [38]. There are two forms of transferrin in birds: ovotransferrin, found in oviducts, and serum transferrin, secreted by the liver. Serum transferrin may stimulate cell proliferation and is regulated by iron levels, while ovotransferrin has a bacteriostatic role independent of iron levels [39,40]. Furthermore, the *TF* has a function in endochondral ossification, being considered the angiogenic molecule released by the hypertrophic cartilage [41]. *TF* presented strong interactions with the *HBG2* (*Hemoglobin Subunit Gamma 2)* gene, which is involved in the transport of oxygen from the lungs to the peripheral tissues, where the beta chain is a component of adult hemoglobin A and D [8]. *HBG2*, as the *TF*, was upregulated in the FHS-affected broilers (Table 3). The gamma globin genes (*HBG1* and *HBG2*) are expressed in several tissues, such as fetal liver, spleen and bone marrow [42]. Chicken has multiple types of globins, and erythropoiesis occurs in two waves: one primitive that acts in blood cells during early embryonic development, and the other producing definitive erythrocytes in late embryonic and post-hatching development [43]. In the bone and cartilage, globin function is not clear, and no information is available on its involvement with FHS in chickens.

The *SLC25A37* (*Solute Carrier Family 25 Member 37*) works as a solute transporter located in the internal mitochondrial membrane, importing iron, which is essential for the synthesis of mitochondrial heme and iron–sulfur clusters [44]. In general, solute carrier proteins are essential to transport a wide set of molecules, such as ions, amino acids and vitamins to the tissues. Recently, it has been demonstrated that they are sensitive to extracellular levels of phosphate [45] and also regulate the pH of the cells [46]. In humans, several bone morphogenic proteins such as *BMP13*, *BMP14* and *BMP15* were responsible for regulating genes of the SLC family [47].

The *ANK1* gene encodes a binding protein of the cytoskeleton, helping to bind other membrane proteins to the actin–spectrin cytoskeleton [48]. It also acts on contact activation, maintenance and proliferation of specialized membrane domains [48]. Multiple ankyrin isoforms with different affinities for various target proteins are expressed in a tissue-specific manner, regulated by development. The *ANK1* is usually found in erythrocytes, but it has already been found in the brain and muscles [49]. In our study, the high expression of *ANK1* can possibly affect the actin structure of the cytoskeleton, changing the structural integrity of the femoral articular cartilage and contributing to the occurrence of FHS. In addition, its expression is related to cell damage, which can be a consequence of FHS, because when FHS starts, positive regulation can act as a sign of attempt to combat the progression of this condition. This gene is also involved in the upregulation of inflammatory cytokines in osteoarthritic lesions [50]. The *ANK1* gene is regulated by the *EPB42*, another gene that was upregulated in the FHS-affected group. The *ANK1*, *EPB42* and *RHAG* (Figure 2) are closely related to some genes that have SNPs differing between the two analyzed groups, such as *TMED2*, *DDX55* (Figure 7) and *POLRC1*.

The *MKNK2* (Serine/threonine-protein kinase 2) gene interacts with MAP kinase and may respond to environmental stress and cytokines. Among its related pathways are the interleukin 1 signaling pathway. MAP/ERK signaling acts as a mediator of the suppressive effects of IFN-gamma in hematopoiesis [51]. The *MKNK2* is not normally expressed in rats, but appears in stressful situations, showing its adaptive function and can also be a signal for cellular apoptosis [52]. Bringing it to our study, this gene may be involved in the initial phase of FHN with the function of initial signaling.

Another DE gene, the *MYH15*, has also been associated with the adrenergic signaling process in cardiomyocytes, in cases of imbalance in the O_2_ supply and removal of CO_2_ from tissues [53]. This gene could be associated with FHS as a consequence of local inflammation.

The *EPX* gene is released during the immune response by eosinophils, having a cytotoxic effect on cells [54,55] and bactericidal activity [56]. The eosinophil peroxidase, an extremely cytotoxic molecule that has anti-inflammatory and pro-inflammatory properties, regulates inflammation by combat invading microorganisms [57,58]. In chickens, the *EPX* gene has been studied as a biomarker for inflammatory events in the gastrointestinal tract [59] and has been related to leg disorders [15,17]. The *EPX* upregulation in broilers affected with FHS may indicate the immune system response to inflammation and, in severe cases, may be related to local necrosis.

The extracellular matrix (ECM) is a stable structural component that is located under the epithelium and close to connective tissue [60], and is responsible in providing support to the tissues and organs throughout the body. It acts in biochemical processes of the body assisting in the differentiation, morphogenesis and homeostasis of tissues [61]. The upregulated genes *ADA* and *RHAG* also participate in the process of metabolic glycosaminoglycans (GAGs) and aminoglycans involved in the metabolism of ECM [62], indicating that the body tries to repair the damage caused by FHS through tissue remodeling. Furthermore, the *ADA* enzyme acts as an endogenous regulator of the adaptive immune system, especially on proliferation and differentiation of T lymphocytes, regulating cell metabolism and triggering several physiological effects on cell proliferation [63,64].

Inflammation is an essential component of the immune system; however, an excess of inflammation can cause tissue damage [65]. The *ADA* gene also acts as a sensor providing information to the immune system regarding tissue damage, protecting cells from excessive tissue damage associated with inflammation [66]. The upregulation of *ADA* has a regulatory role in immune responses, acting on the activation and regulation of lymphocyte and neutrophil levels [64,67]. In our study, the *ADA* was upregulated in AC and GP in the FHS-affected group (Table 3), possibly due to the large tissue inflammation that occurs in the affected animals. The presence of specific variants in this gene between the studied groups also highlights that those genetic mechanisms could be involved with FHS in chickens. The *ADA* gene is in a branch of the gene network with several genes that have SNPs with different genotypes between the normal and affected groups, such as *TRIM25*, *RPS24*, *NACAD*, and others (Figure 7). The *TRIM25* and *ADA* are important genes to the RNA machinery [66,68], and the involvement of the *ADA* in bone metabolism has been observed in humans [69]. Deficiency in *ADA* expression has led to a reduction of bone volume and downregulation of *RANKL* expression, while *ADA* upregulation can lead to skeletal abnormalities such as scapular spurring [70,71].

The *ADA* and *IFI6* genes also play an important role in the regulation of apoptosis, which is an essential physiological mechanism in the development and tissue homeostasis [14,72]. The *IFI6* gene, also known as *ISG12*, regulates cellular metabolism during the differentiation of osteoblasts and apoptosis [14,73]. The upregulation of the *IFI6* gene may be related to a causative factor, stimulating apoptosis in the articular cartilage, leading the animal to be more susceptible to FHN.

Furthermore, once the exclusively DE genes in the GP were evaluated, the main biological processes were those related to chondrocyte differentiation and cartilage development, grouping three main genes that had the lowest expression in the affected group: *CHADL*, *GDF5* and *PTHLH* (Figure 6, Table S5). Chondroadherin-like (*CHADL)* is a member of a family of collagen-associated small leucine-rich proteins (SLRPs) responsible for signaling of differentially regulated collagen fibrils during development, homeostasis, or pathogenesis. *CHADL* is expressed in cartilaginous tissues, influences collagen fibrillogenesis and negatively modulates chondrocyte differentiation [74].

In humans, increased *GDF5* gene expression is related to joint tissue remodeling. After joint damage, its high expression in chondrocytes is observed, both in the new cartilage recovery tissue and in the adjacent damaged cartilage [75]. The *PTHLH* gene promotes the proliferation of chondrocytes and inhibits their hypertrophy and terminal differentiation. In patients with osteoarthritis, this gene is highly expressed in chondrocytes and synovial fluid [76].

Among the BP of the AC, the response against bacteria can be highlighted, with all genes involved in this BP being more expressed in the FHS-affected group (Figure 1). Among them, cathelicidin-2 (*CAMP*) and cathelicidin-3 (*CATH3*) stand out, which have a broad spectrum of antimicrobial activity and the ability to limit inflammation by inhibiting the activation of Toll-like receptor 2 (*TLR2*) and *TLR4* [77].

These results suggest that the growth plate is not fully efficient in the process of recovery and remodeling of damaged cartilage. Conversely, articular cartilage can partially control bacterial infection and the intensity of inflammation that can intensify tissue damage. Other mechanisms should be evoked.

In addition to the identification of the DE genes, biological processes and SNPs found in our study, another interesting result presented here was the comparison of the chicken transcriptomes with other datasets. When we compared the DE genes obtained in the AC and GP tissues with other gene expression datasets obtained in the GEO, we found four genes in common, *SLC4A1* (Solute Carrier Family 4 Member 1), *EPB42* (Erythrocyte Membrane Protein Band 4.2), *SPTB* (Spectrin Beta. Erythrocytic) and *ANK1* (Ankyrin 1) (Figure 6A), all of them related to erythrocyte and ion transport functions. In the second comparison, using the DE genes of bone and cartilage datasets, besides the four previously described genes (*SLC4A1*, *EPB42*, *ANK1* and *SPTB)*, four additional genes were found (*CCL26*, *ADA*, *SLC25A37* and *MKNK2)*, all involved with immune response. Furthermore, two genes, the *CCL26* and *SLC4A1*, have not been enriched in the gene network. *CCL26* (chemokine (C-C motif) ligand 26) is from the cytokine family of secreted proteins involved in immunoregulatory and inflammatory processes, displaying chemotactic activity for normal peripheral blood eosinophils and basophils, and it may contribute to the eosinophil accumulation in atopic diseases. Finally, the *SLC4A1* gene encodes a protein that is part of the anion exchanger family and is expressed in the plasma membrane of erythrocytes, involved in the transport of carbon dioxide from the tissues to the lungs. Furthermore, this gene is located in QTL regions previously described as associated with femur mineral content and femur weight, which reinforces its importance as a good candidate gene to FHS.

Based on the functions and the high expression of the genes that were DE in both tissues, we had two hypotheses: the first is that the high expression of these genes may be a consequence of the FHS, due to cell damage and from the attempt to combat the progression of this condition. The second hypothesis is that the high expression and possible mutations of these genes cause FHN, as they can alter the structure of the actin cytoskeleton, affecting the structural integrity of the femoral articular cartilage, contributing to the occurrence of FHS. Therefore, using the two datasets in this study, we could highlight common genes that were regulated in the two tissues, as well as those that were exclusively DE in each one. With this information, new genes involved with FHS in broilers were described, where most of them have never been cited as candidates to this condition in chickens. We have also shown conserved mechanisms among huma and chicken osteonecrosis. Furthermore, genetic variants were identified; some of them were described for the first time in this study, and some of them differed between the normal and FHS-affected groups and could be further investigated as molecular markers for this condition. Finally, our results can also contribute to understand the onset of this condition in other species since the molecular mechanisms seem to be at least in part conserved among them.

## 5. Conclusions

With the integrated analysis of gene expression of the two tissues and variant identification, we were able to detect strong candidate genes and SNPs related to FHS/FHN in chickens. Comparing the AC and GP DE genes with the other datasets related to FHN allowed us to identify genes possibly involved with FHN, evincing the shared mechanisms across different species. Finally, the role of *SLC4A1*, *RHAG*, *ANK1*, *MKNK2*, *SPTB*, *ADA*, *C7* and *EPB420* genes should be more explored in order to validate them as candidates for FHS/FHN.

## Figures and Tables

**Table 1:** Primers used in the qPCR analysis of articular cartilage and growth plate of normal and FHS-affected broilers.

Gene	Ensembl ID	Primer (5′ to 3′)
***C7***	ENSGALG00000014835	F: GTGGTCCTTCCCTGGACATC
Gallus gallus complement C7		R: GTTGGATCGCGCTTCACATT
***EPB42***	ENSGALG00000021230	F: GCTACAGACCTGGTACTTGGA
Gallus gallus erythrocyte membrane protein band 4.2		R: GACTTGCAGGGTTCTAACTTCG
***MKNK2***	ENSGALG00000003845	F: CATCCTGCAGAGGAACAGCA
Gallus gallus MAPK interacting serine/threonine kinase 2		R: CCTGGAGGCTGCTTTGATGA
***SLC4A1***	ENSGALG00000039978	F:TCGAGCTCAAATGCCTGGAC
Gallus gallus solute carrier family 4 member 1 (Diego blood group)		R: GGCATCTGCACCTCGTTGTA
***SPTB***	ENSGALG00000036805	F: CTGGATGCTGATGAGGCCAA
Gallus gallus spectrin beta, erythrocytic		R: CTTTCCTCGTCCTTCGGCTT
***ANK1*** *	ENSGALG00000029534	F: CCACCATCCCACCATTCAGT
Ankyrin 1		R: ACGGTCACAAACTCCAGCAT
***ADA*** *	ENSGALG00000004170	F:TTCGGCAAGAAAAGAGGGGT
adenosine deaminase		R: GTGTTTGGTAGCTGACGTGC
***RHAG*** *	ENSGALG00000016684	F:TCTGGAGATCACGGCCTTTG
Rh associated glycoprotein		R:GCTCCAATATCTGTGGCCTGA

* Hul et al. [17].

**Table 2:** Characterization of the transcripts identified in the articular cartilage (AC) and femoral growth plate (GP) tissues.

	AC		GP	
Annotated Transcripts	Transcript Number	%	Transcript Number	%
IG_V_gene	3	0.02%	20	0.16%
*LncRNA*	413	3.40%	454	3.59%
*MiRNA*	20	0.16%	22	0.17%
*misc_RNA*	1	0.01%	2	0.02%
*Mt_Rrna*	2	0.02%	2	0.02%
protein_coding	11,577	95.03%	11,942	94.54%
Pseudogene	155	1.27%	162	1.28%
*rRNA*	1	0.01%	0	0
*scaRNA*	1	0.01%	0	0
*snoRNA*	8	0.07%	26	0.21%
*snRNA*	1	0.01%	1	0.01%
*sRNA*			1	0.01%
Total annotated transcripts	12,182		12,632	

**Table 3:** Common differentially expressed genes between articular cartilage (AC) and femoral growth plate (GP) with information of the Ensembl ID, gene name, log2 fold change (logFC) and false discovery rate (FDR).

		AC		GP	
Ensembl ID	Gene Name	logFC	FDR	logFC	FDR
ENSGALG00000043254	*EPX*	4.25	4.11 × 10^−9^	2.59	0.0009
ENSGALG00000003212	*TSPO2*	3.66	7.20 × 10^−5^	1.54	0.005
ENSGALG00000040279	*RHCE*	2.02	0.0008	1.29	0.002
ENSGALG00000026518	*RUNDC3A*	2.44	0.003	1.10	0.02
ENSGALG00000039978	*SLC4A1*	2.70	0.003	1.20	0.01
ENSGALG00000007447	*TUBB1*	1.99	0.003	1.28	0.01
ENSGALG00000011190	*PLACL2*	2.54	0.005	1.10	0.0002
ENSGALG00000042105	*PLCB2*	1.81	0.005	0.67	0.03
ENSGALG00000014585	*CCL26*	2.36	0.008	1.12	0.04
ENSGALG00000021230	*EPB42*	2.69	0.008	1.33	0.02
ENSGALG00000004170	*ADA*	1.71	0.008	1.01	0.001
ENSGALG00000015358	*MYH15*	−1.91	0.01	−1.38	0.03
ENSGALG00000016684	*RHAG*	2.43	0.01	1.34	0.006
ENSGALG00000029857	*GIMAP6*	1.79	0.01	0.76	0.01
ENSGALG00000013575	*IFI6*	2.87	0.01	2.22	0.01
ENSGALG00000009479	*SAMD9L*	3.10	0.01	1.78	0.01
ENSGALG00000000378	*SLC25A37*	1.76	0.01	1.32	0.001
ENSGALG00000041693		2.81	0.01	1.33	0.04
ENSGALG00000003845	*MKNK2*	1.40	0.01	0.63	0.009
ENSGALG00000026948	*ADD2*	2.12	0.01	1.27	1.85
ENSGALG00000036805	*SPTB*	2.19	0.01	1.09	0.005
ENSGALG00000051068		2.38	0.02	1.41	0.0005
ENSGALG00000003594	*ANK1*	2.11	0.02	0.99	0.01
ENSGALG00000014736	*KEL*	1.99	0.02	1.21	0.005
ENSGALG00000043671		−1.24	0.02	−1.03	0.02
ENSGALG00000028273	*HBE1*	3.74	0.02	2.24	0.04
ENSGALG00000039269	*RNF213*	2.06	0.027	1.06	0.03
ENSGALG00000014835	*C7*	2.84	0.027	1.55	0.001
ENSGALG00000028357	*LOC428421*	2.50	0.036	1.49	0.006
ENSGALG00000002192	*PTPRC*	1.22	0.037	0.61	0.03
ENSGALG00000045776	*CPN2*	1.77	0.037	0.90	0.02
ENSGALG00000014686	*FBN2*	−1.08	0.037	−1.72	0.0005
ENSGALG00000030247	*TMOD4*	2.05	0.04	1.06	0.01
ENSGALG00000009552	*LOC423277*	1.78	0.04	1.14	0.04
ENSGALG00000044326	*LOC426820*	1.54	0.04	0.80	0.01
ENSGALG00000006453	*TF*	3.04	0.04	0.67	0.04

## Data Availability

The datasets used or analyzed during the current study are available from the corresponding author on reasonable request. The transcriptome sequences are available in the SRA database with BioProject number PRJNA352962 and PRJNA350521.

## Supplementary Materials

The following supporting information can be downloaded at: https://www.mdpi.com/article/10.3390/ani12060788/s1, Supplementary File S1: Table S1. Input paired read numbers, reads number after quality control and mapping information of each sample. A table containing the number of raw reads and after quality control and mapping information. Table S2. Expressed and DE genes between normal and FHS-affected broilers with 35 days of age in the AC. An excel sheet with the expressed and DE genes between normal and FHS-affected broilers with 35 days of age. Table S3. Expressed and DE genes between normal and FHS-affected broilers with 35 days of age in the GP. An excel sheet with the expressed and DE genes between normal and FHS-affected broilers with 35 days of age. Table S4. Main biological processes involved with FHS considering the DE genes exclusive. Table S5. Main biological processes involved with FHS considering the DE genes exclusive in the growth plate using the DAVID database. Table S6. Variants identified between normal and FHS-affected broilers with 35 days of age in the AC and GP transcriptomes according to the location in the Gallus gallus reference genome (GRCg6). Table S7. Variants annotated by the VEP tool (Ensembl 103). Table S8. Variants annotated in QTL regions using the VEP tool (Ensembl 103). Table S9. Candidate genes in human QTLs according to EnrichR. The genes identified in the common differentially expressed and variant analysis were used to verify their presence in human GWAS. Supplementary File S2: Figure S1. Heatmap gene cluster classification for AC and GP normal and FHS-affected samples. In the heatmap, the expression for each gene is presented in the rows, and samples are visualized in the columns, showing a hierarchical clustering of genes and samples. In red, DE genes were downregulated, and in green, upregulated in the affected samples.
