# Supplementary material for: Comprehensive Analyses of Bone and Cartilage Transcriptomes Evince Ion Transport, Inflammation and Cartilage Development-Related Genes Involved in Chickens’ Femoral Head Separation
Source: Animals (Basel). 2022 Mar 20;12(6):788. doi: 10.3390/ani12060788 (PMC8944783; doi:10.3390/ani12060788)
Supplement: Supplementary file 1 [file animals-12-00788-s001.zip › Supplementary_file_S2.pdf]

Comprehensive analyses of bone and cartilage transcriptomes evince ion transport, inflammation and cartilage development-related genes involved in chickens' femoral head separation

Iara Goldoni, Adriana Mércia Guaratini Ibelli, Lana Teixeira Fernandes, Jane de Oliveira Peixoto, Maurício Egídio Cantão, Mônica Corrêa Ledur

Additional file S2.

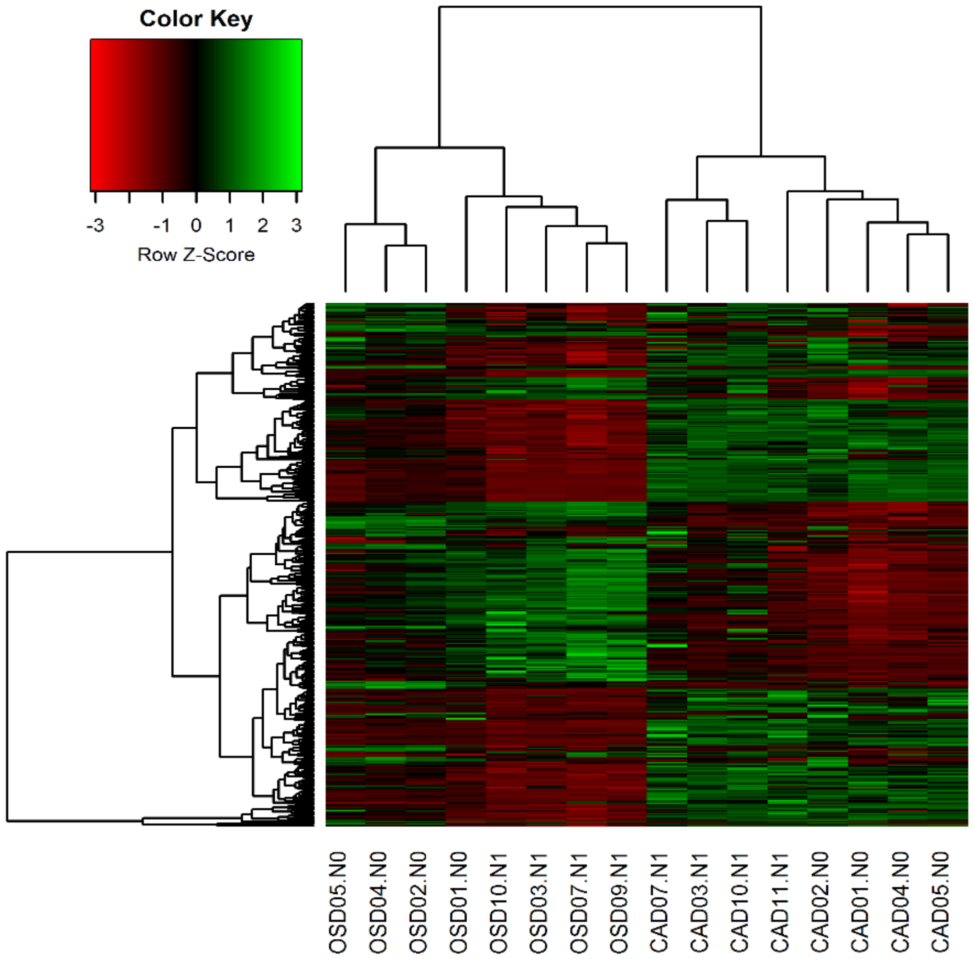

Fig S1. Heatmap gene cluster classification for AC and GP normal and FHS-affected samples.

In the heatmap, the expression for each gene is presented in the rows and sample is visualized

in the columns, showing a hierarchical clustering of genes and samples. In red, DE genes were downregulated and in green, upregulate in the affected samples.
